# Identification of c-MYC SUMOylation by Mass Spectrometry

**DOI:** 10.1371/journal.pone.0115337

**Published:** 2014-12-18

**Authors:** Manpreet Kalkat, Pak-Kei Chan, Amanda R. Wasylishen, Tharan Srikumar, Sam S. Kim, Romina Ponzielli, David P. Bazett-Jones, Brian Raught, Linda Z. Penn

**Affiliations:** 1 Princess Margaret Cancer Centre, University Health Network, Toronto, Canada; 2 Department of Medical Biophysics, University of Toronto, Toronto, Canada; 3 Program in Genetics and Genome Biology, The Hospital for Sick Children, Toronto, Canada; California Institute of Technology, United States of America

## Abstract

The c-MYC transcription factor is a master regulator of many cellular processes and deregulation of this oncogene has been linked to more than 50% of all cancers. This deregulation can take many forms, including altered post-translational regulation. Here, using immunoprecipitation combined with mass spectrometry, we identified a MYC SUMOylation site (K326). Abrogation of signaling through this residue by substitution with arginine (K326R) has no obvious effects on MYC half-life, intracellular localization, transcriptional targets, nor on the biological effects of MYC overexpression in two different cell systems assessed for soft agar colony formation, proliferation, and apoptosis. While we have definitively demonstrated that MYC SUMOylation can occur on K326, future work will be needed to elucidate the mechanisms and biological significance of MYC regulation by SUMOylation.

## Introduction

c-MYC (MYC) is a DNA binding transcription factor and a master regulator of many cellular functions. For example, MYC can induce cellular proliferation, growth and biomass accumulation and is frequently deregulated in human malignancies to further drive the pro-growth state of a tumor cell [1]. In normal cells, MYC is tightly controlled at a number of steps, including at the transcriptional, translational and post-translational levels. Altered regulation at any of these steps can result in deregulated, oncogenic MYC [2]. One well-studied canonical pathway that is known to regulate MYC activity and stability at the post-translational level is the GSK3 pathway [2]. The GSK3-FBXW7 axis regulates MYC via phosphorylation at T58, followed by ubiquitylation of MYC by the E3 ubiquitin ligase complex SCF-FBXW7 and subsequent proteasomal degradation [3]–[5]. Accordingly, substituting threonine 58 with alanine (T58A) confers increased stability and transformative potential [5]–[7]. Thus, characterizing the post-translational modifications (PTMs) of MYC can lead to a better understanding of the regulatory mechanisms controlling this potent oncogene.

SUMOylation is a post-translational modification that utilizes a series of E1, E2 and E3 proteins for conjugation of a small ubiquitin-like modifier (SUMO) moiety to its target protein [8]. In mammalian cells, three SUMO protein paralogs are expressed, all of which are capable of covalently modifying substrates. SUMO2 and 3 are more than 90% identical in sequence, while SUMO1 shares ∼50% homology with these two proteins [9]. Growing evidence indicates that SUMOylation has many important roles in the cell, such as response to cellular stressors [10], [11] and transcriptional regulation [12], [13]. Transcriptional regulators are frequently SUMOylated, leading to alterations in their activity [14], [15]. Moreover, recent reports have unveiled a potential role for SUMOylation in MYC-driven tumorigenesis [16]. Specifically, in a modified synthetic lethal screen, inducible MYC expression was found to lead to a reliance on the SUMO system for cell survival [16]. Furthermore, MYC can transcriptionally induce a component of the SUMO E1, SUMO activating enzyme subunit 1 (SAE1) [17].

Herein, by performing dual-immunoprecipitation coupled with mass spectrometry, we provide definitive evidence that MYC is SUMOylated at K326, corroborating a recent study that was published during the course of our work suggesting MYC SUMOylation at K323 and/or K326 [18]. Abrogation of signaling through this residue, as assessed by a lysine to arginine mutant (K326R) tested in a number of different biological assays and cell lines, does not result in any significant changes to MYC activity.

## Materials and Methods

### Cell Lines

SH-EP/TET21/N-MYC (SH-EP) cells were a kind gift of Manfred Schwab [19]. MCF10A cells were a kind gift of Senthil Muthuswamy [20]. SH-EP, MCF10A and 293Tv cells were cultured as previously described [20]–[22]. Transgene expression was stably introduced into SH-EP cells using retroviral insertion with pMN-ires-GFP and cells were flow-sorted for GFP positivity. SH-EP cells were treated with 1 µg/mL doxycycline (Sigma) to repress N-Myc expression 24 hours prior to each experiment. MCF10A and 293Tv cells were infected with lentiviral particles encoding GEV16 and pF 5xUAS and selected for hygromycin (Bioshop) and puromycin (Sigma) resistance respectively.

### Immunoblotting

Lysates were prepared from subconfluent cells harvested directly in boiling SDS lysis buffer (1% SDS, 11% glycerol, 10% β-mercaptoethanol, 0.1 M Tris pH 6.8) and boiled prior to SDS-PAGE. The following antibodies were used for detection: mouse monoclonal anti-c-MYC 9E10 (1∶1000, prepared in-house), rabbit polyclonal anti-c-MYC (1∶1000, Millipore #06-340), mouse monoclonal anti-Flag M2 (1∶1000, Sigma #3165), rabbit polyclonal anti-actin (1∶2500, Sigma #A2066), rabbit polyclonal SUMO1 (1∶1000, Abcam #ab32058), rabbit polyclonal SUMO2/3 (1∶1000, Abcam #ab3742), rabbit polyclonal anti-N-Myc (1∶500, Santa Cruz #sc-791). Primary antibodies were detected using IRDye-labeled secondary antibodies (1∶20000, LI-COR Biosciences) or HRP-conjugated secondary antibodies (1∶10000, GE Healthcare). For MG132 treatments, cells were treated with 10 µM MG132 (Calbiochem), diluted from a stock solution of 50 mM dissolved in DMSO for 4 hours prior to harvest.

### Cellular Fractionations

Cells were lysed in 1 mL of Buffer A (10 mM HEPES pH 7.9, 10 mM KCL, 0.1 mM EDTA, 0.1 mM EGTA, 1 mM DTT, 0.5 µg/mL antipain, 1 µg/mL leupeptin, 1 µg/mL aprotinin, 1 µg/mL pepstatin) and incubated on ice for 15 minutes. NP-40 was added to a final concentration of 0.5% and the lysate was centrifuged for 5 minutes at 956 *g* at 4°C. The resulting supernatant is the cytosolic compartment. The nuclear pellet was washed with Buffer A and then lysed in SDS lysis buffer. Sample buffer was added to both cytosolic and nuclear extracts and boiled prior to SDS-PAGE. Rabbit polyclonal anti-lamin B1 (1∶3000, Abcam #ab16048) was used as a nuclear marker, and mouse monoclonal anti-tubulin (1∶2000, Calbiochem #DM1A) was used as a cytosolic marker. c-MYC was visualized using rabbit polyclonal anti-c-MYC antibody (1∶1000, Millipore #06-340).

### Dual Immunoprecipitation and Mass Spectrometry

Cells were lysed in 1% SDS lysis buffer and diluted 1∶10 prior to addition of antibody (anti-MYC N-262, prepared in-house) and G beads (GE Healthcare) for overnight incubation at 4°C. Beads were washed 3x with PBS and eluted into 2xSDS lysis buffer. The eluate was diluted to 0.1% SDS content and an overnight immunoprecipitation was conducted with anti-Flag M2 affinity gel (Sigma #A2220). Mass spectrometry was performed as previously described [23] and data analyzed using SUMmOn software [24].

### Immunoprecipitation (IP) and Western Blotting

Cells were lysed in boiling SDS lysis buffer. Samples were syringed with a 25-gauge needle and centrifuged at 20817 *g* for 5 minutes. The resulting supernatant was diluted to 0.1% SDS content with IP dilution buffer (10 mM N-Ethylmaleimide, 0.5 µg/mL antipain, 1 µg/mL leupeptin, 1 µg/mL aprotinin, 1 µg/mL pepstatin in PBS) and immunoprecipitated with purified anti-c-MYC 9E10 antibody in the presence of 250 units turbonuclease (BioVision) per IP rotating overnight at 4°C. G sepharose beads (GE Healthcare) were washed with PBS and were incubated with the sample for 1 hour. The beads were then washed 3x with PBS and the sample was eluted using 4x SDS loading dye and subjected to SDS-PAGE.

### Co-immunoprecipitation and Western Blotting

Cells were lysed in Buffer B (20 mM HEPES pH 7.9, 400 mM NaCl, 1 mM EDTA, 1 mM EGTA, 1 mM DTT, 0.5 µg/mL antipain, 1 µg/mL leupeptin, 1 µg/mL aprotinin, 1 µg/mL pepstatin) by incubating on ice for 15 minutes. NP-40 was added to a final concentration of 0.5% and the samples were centrifuged for 5 minutes at 956 *g* at 4°C. The samples were diluted 1∶1 with Buffer C (20 mM HEPES pH 7.9, 1 mM EDTA, 1 mM EGTA, 0.5 µg/mL antipain, 1 µg/mL leupeptin, 1 µg/mL aprotinin, 1 µg/mL pepstatin) and incubated with either anti-MAX antibody (#C-124, Santa Cruz) or normal rabbit IgG (Santa Cruz). G beads were washed with a 1∶1 mix of Buffer B and C and then incubated with the samples for 1 hour. The beads were then washed 3x with a 1∶1 mix of Buffer B and C and samples were eluted with 4x SDS loading dye and subjected to SDS-PAGE. MYC was visualized using anti-c-MYC 9E10 (1∶1000).

### Soft Agar Colony Formation Assay

Transformation was evaluated by anchorage-independent colony growth as previously described [21], [23].

### Mutagenesis

K326R mutation was generated in pMN-MYC-ires-GFP and pcDNA3-MYC background by site-directed mutagenesis using the QuikChange Site-Directed Mutagenesis Kit (Agilent Technologies) according to manufacturer's protocol using the following primers: 5'-GCCAAGAGGGTCAGGTTGGACAGTGTC-3' and 5-GACACTGTCCAACCTGACCCTCTTGGC-3'. pcDNA3-K326R was excised and inserted into the pF 5xUAS backbone[25] using BamH1 (New England Biolabs) and Xba1 (New England Biolabs) restriction sites.

### EdU Incorporation

MCF10A cells were seeded at a density of 250 000 cells per 10 cm dish and arrested in G1 using starvation media (DMEM/HAMF12 1∶1 mix supplemented with 0.05% horse serum and 10 µg/mL insulin (Sigma)) for 24 hours. 10 µg/mL ethynyl-2′-deoxyuridine (EdU), a thymidine analog, was then added along with 100 nM 4-hydroxytamoxifen (4-OHT, Sigma). Cells were harvested after 24 hours and staining for EdU incorporation was performed according to manufacturer's instructions (Click-It Edu Alexa Fluor 488 Imaging Kit, Life Technologies).

### Cycloheximide Half-Life Experiments

MCF10A cells were seeded at 60% confluence on 6 cm dishes and treated with 100 nM 4-OHT for 24 hours. Cells were then treated with 10 µg/mL cycloheximide (Sigma) and protein lysates were collected every 15 minutes for eight timepoints. Lysates were subjected to SDS-PAGE and blotted for MYC expression with actin used as a loading control. Primary antibodies were detected using IRDye-labeled secondary antibodies (1∶20000, LI-COR Biosciences). MYC expression was quantified relative to actin using ImageJ Software (NIH) and Graphpad Prism Software was used to calculate half-life values.

### Proliferation Assay

SH-EP cells were seeded at 500 cells/well in 96 well format in triplicate. CyQuant NF reagent (Invitrogen) was used according to manufacturer's instructions to quantify DNA content on days 0 through 4. Data was normalized to day 0 seeding densities to obtain relative cell numbers. Doubling time was calculated using GraphPad Prism Software.

### Cell Death Assay

MCF10A cells were seeded at a density of 250 000 cells per 10 cm dish and arrested in G1 using starvation media (DMEM/HAMF12 1∶1 mix supplemented with 0.05% horse serum and 10 µg/mL insulin) for 24 hours. Cells were then treated with 100 nM 4-OHT and 500 ng/mL tunicamycin (Sigma) for an additional 24 hours prior to fixation. Cells were stained with propidium iodide (Sigma) and analyzed for pre-G1 content by flow-cytometry as previously described [26].

### Dual Luciferase Experiments

Luciferase reporter assays were performed as previously described [22].

### Immunofluorescence Experiments

Cells were grown on glass coverslips overnight and were either treated with DMSO or MG132 for 4 hours. The samples were fixed with 2% paraformaldehyde (in PBS, pH 7.2) for 20 minutes. Samples were then washed 3x with PBS and blocked with 2% BSA with 0.1% Triton X-100 in PBS for 1 hour. Anti-c-MYC primary antibody (1∶200, Millipore #06-340) was incubated with 1% BSA and 0.05% Triton X-100 in PBS overnight in a humidified chamber. The samples were washed 3x with PBS. Alexa Fluor 568 donkey anti-rabbit (1∶400, Life Technologies) was diluted in 1% donkey serum with 0.05% triton X-100 in PBS and incubated with the samples for 1 hour. Samples were washed 3x with PBS and mounted on glass slides with Duolink *In Situ* mounting medium with DAPI (Sigma). Images were acquired on a Zeiss LSM700 Confocal Microscope.

### Statistical Analyses

All figures are representative of the mean ± standard deviation of a minimum of three biological replicates. Differences between three groups were assessed using a one-way ANOVA with Bonferroni correction using Graphpad Prism Software.

## Results

### Identification of a MYC SUMOylation Site at Lysine 326

**Figure 1:**
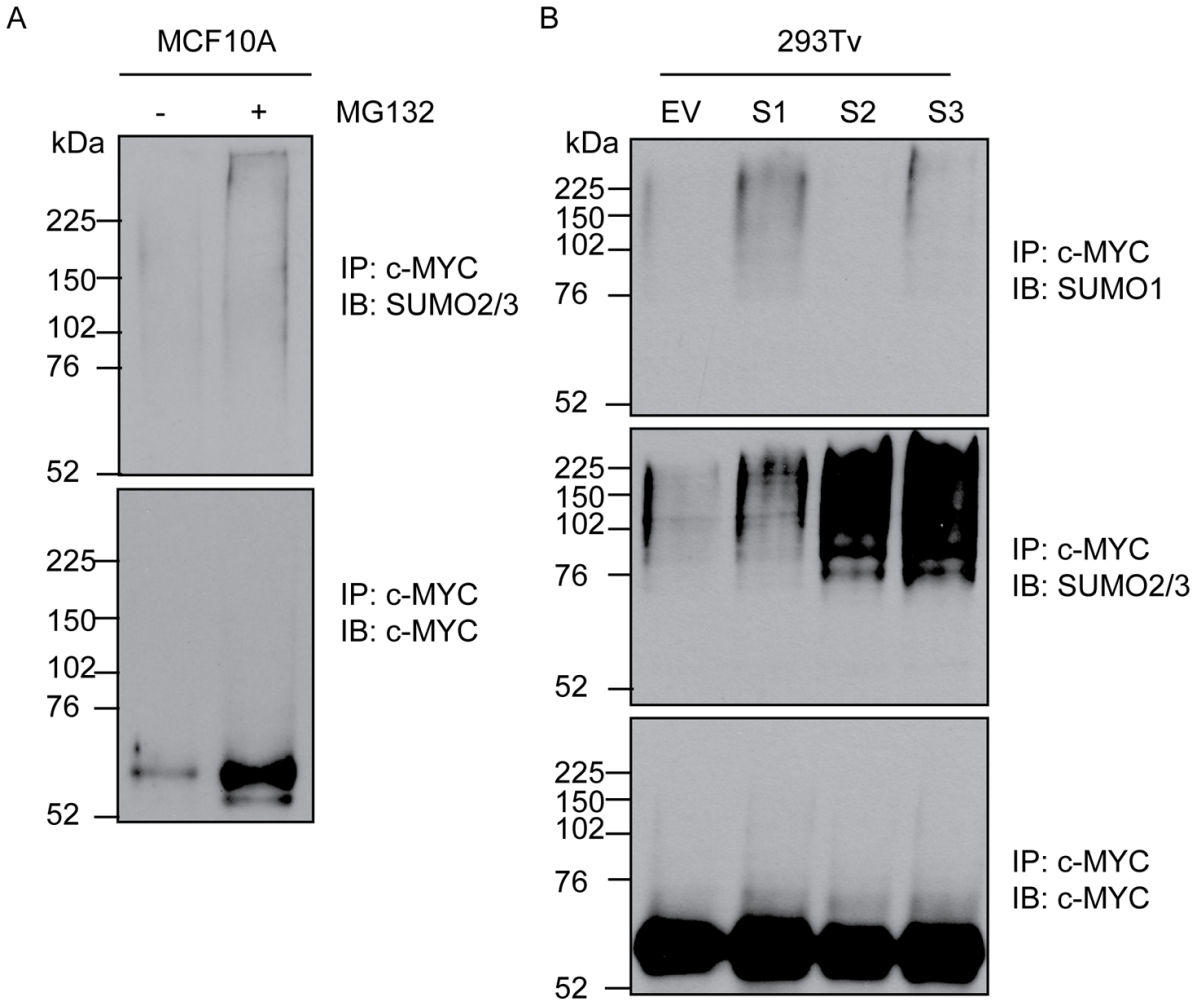
MYC SUMOylation is observed following proteasomal inhibition. (A) MYC was immunoprecipitated under denaturing conditions from samples either treated with vehicle control (DMSO) or MG132 for 4 hours. Immunoblotting for SUMO2/3 revealed the accumulation of high molecular weight SUMO signal following proteasomal inhibition. (B) 293Tv cells were transfected with MYC and either empty vector (EV) control or SUMO paralogs 1–3 (S1, S2, S3). After 24 hours, cells were harvested and subjected to an immunoprecipitation for MYC followed by immunoblotting for the presence of SUMO paralogs.

**Figure 2:**
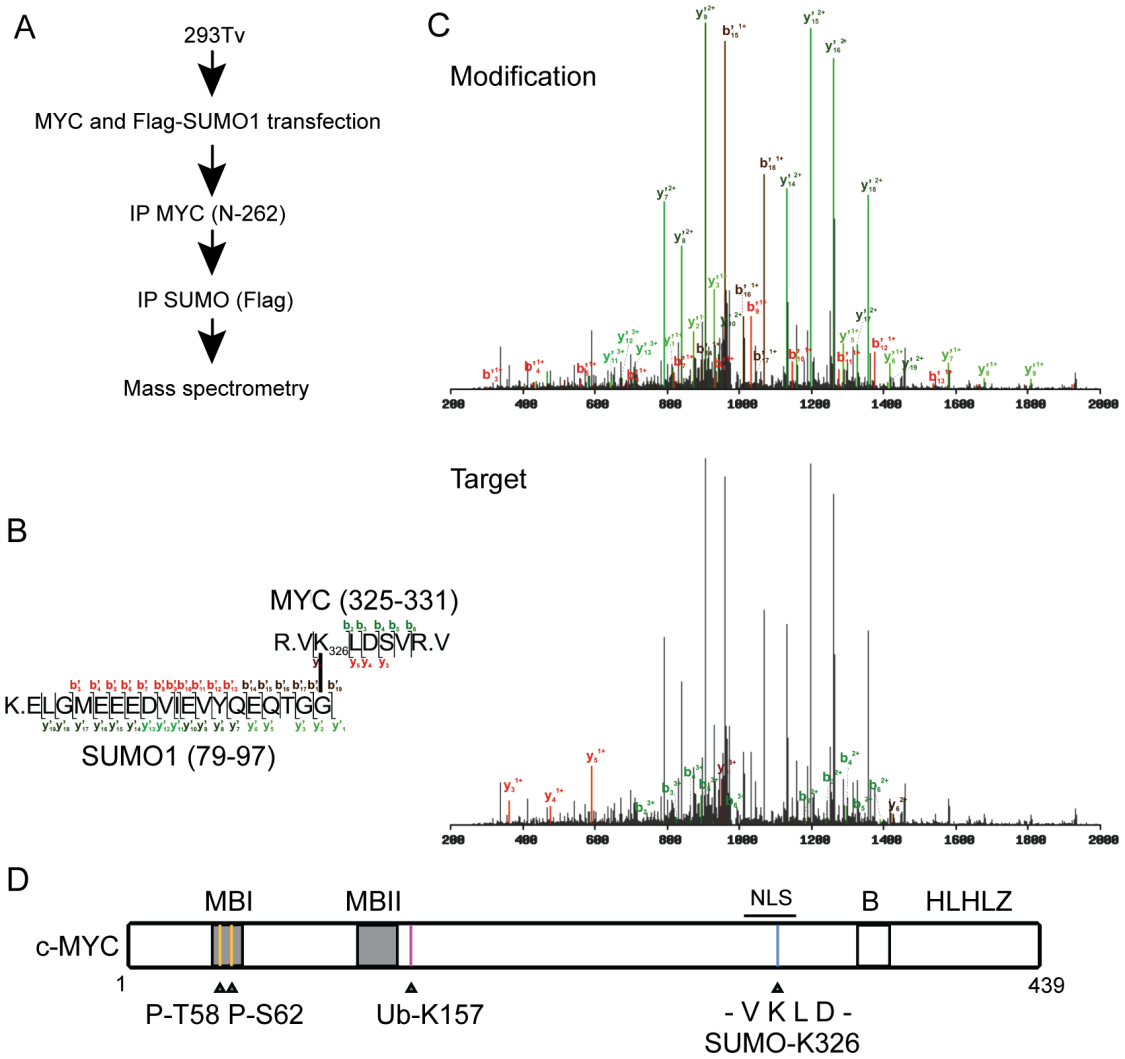
Identification of a SUMO site on MYC. (A) 293Tv cells were transfected with MYC and Flag-SUMO1. Cells were then lysed under denaturing conditions and sequential pulldowns were performed first for MYC and then for FlagSUMO1. The resulting eluate was subjected to mass spectrometry analysis. (B) Observed sequence of the detected peptide fragments for both the modification (SUMO1) and the target (MYC). (C) Mass spectrometry fragmentation spectra for MYC (target) and SUMO (modification) demonstrating MYC SUMOylation at K326. (D) Diagrammatic representation of MYC and the post-translational modifications observed by mass spectrometry. P: phosphorylation, Ub: ubiquitylation, B: basic region, HLH-LZ: helix-loop-helix leucine zipper, NLS: nuclear localization signal. Grey boxes indicate regions of homology among MYC family proteins, termed MYC Boxes (MBs).

SUMOylation of transcription factors can alter their activity, intracellular localization or half-life, usually via alterations in protein-protein interactions [27]. Given the central role of MYC in tumorigenesis, and a recent report highlighting a genetic interaction between MYC and the SUMO pathway [16], we sought to determine if MYC activity could be regulated by SUMOylation. Non-transformed breast epithelial MCF10A cells were treated with the proteasome inhibitor MG132. Upon immunoprecipitation of denatured protein lysates for MYC, an observable signal for SUMO2/3 was detectable in the sample treated with MG132 as compared to DMSO treated cells (Fig. 1A). To then determine if MYC could be modified by SUMO paralogs 1–3, we transiently transfected 293Tv cells with ectopic MYC and either empty vector, SUMO1, SUMO2 or SUMO3 constructs. Immunoprecipitation for MYC followed by immunoblotting with antibodies specific for either SUMO1 or SUMO2/3 revealed an accumulation of SUMO signal with all three paralogs (Fig. 1B). We also observed a fainter signal in MYC transfected cells alone (empty vector, EV). The apparent molecular weight of MYC is approximately 64 kDa. A molecular weight shift of approximately 12 kDa upon SUMO transfection is consistent with the molecular weight of SUMO. The SUMOylation signal extended from the 76 kDa protein weight marker to greater than 250 kDa, suggestive of polySUMOylation. We then sought to determine the sites of MYC SUMOylation. 293Tv cells were transiently transfected with constructs encoding MYC and FlagSUMO1. After 24 hours, cells were lysed under denaturing conditions, the lysate diluted 1∶10 with PBS, then subjected to a dual immunoprecipitation protocol whereby MYC was first isolated using an anti-MYC polyclonal antibody (N-262), eluted, and then the eluate subjected to a second round of immunoprecipitation using anti-Flag antibody (Fig. 2A). The resulting eluate was analyzed by mass spectrometry, identifying a number of peptides corresponding to MYC, ubiquitin and both SUMO1 and SUMO2/3. We were also able to detect previously described modifications on MYC, including phosphorylation of T58 and S62. Ubiquitylation of K157 [28] was confirmed, and K48 ubiquitin chain linkages were identified in the eluate. Furthermore, using SUMmOn analysis [24], [29], we directly observed SUMOylation of MYC on K326 (Fig. 2B,C and S1 Figure). This site maps to a consensus SUMOylation sequence (Fig. 2D).

### K326R Mutant has No Effect on Cell Proliferation and Death in MCF10A Cells

**Figure 3:**
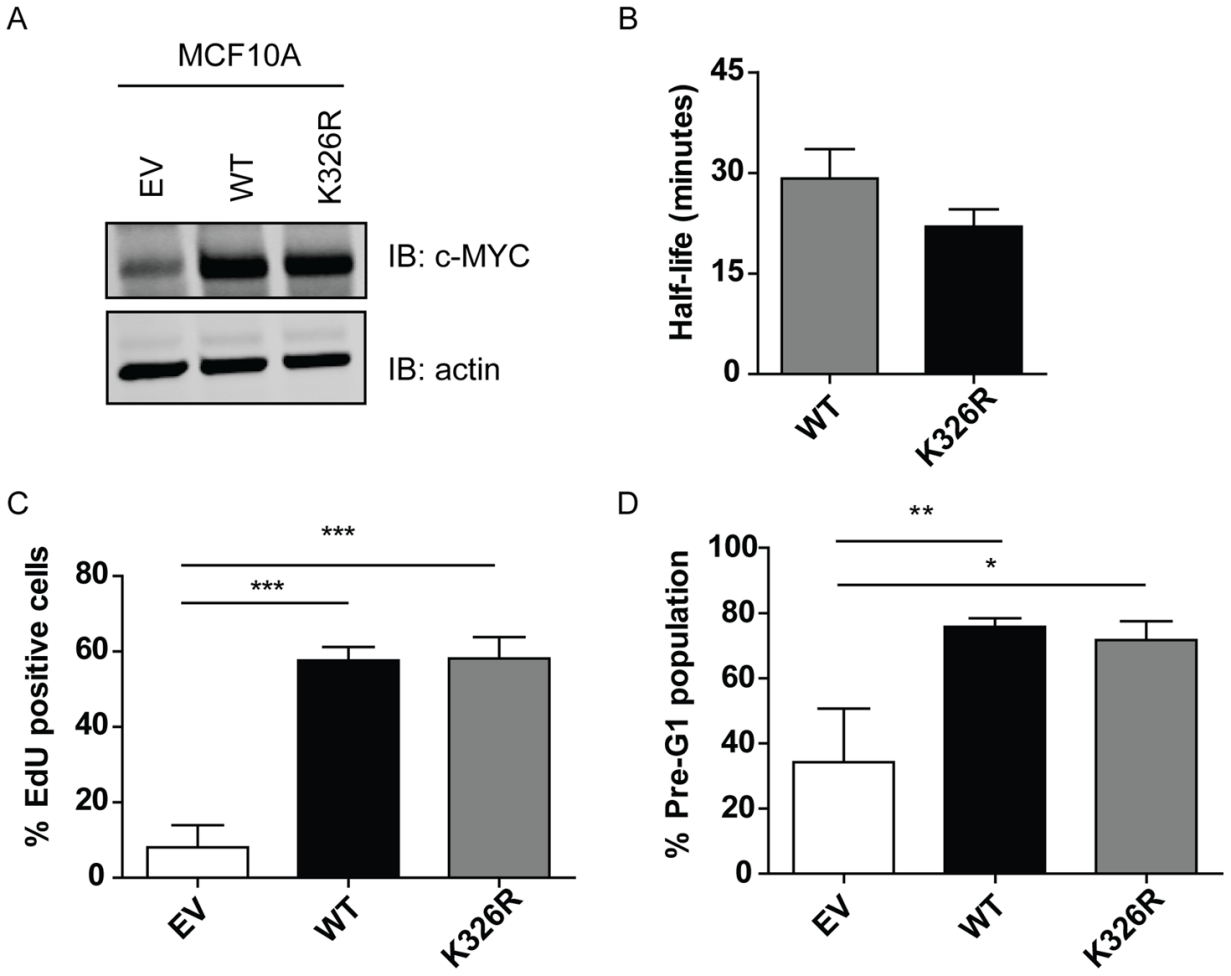
K326R mutant has no effect on cell proliferation and death in MCF10A cells. (A) Western blot depicting the relative expression levels of MYC in empty vector control (EV), wild-type (WT) and K326R cells following 24 hours of 4-hydroxytamoxifen (4OHT) treatment. (B) MCF10A cells were treated with 4OHT to induce transgene expression. 24 hours post-treatment, cells were treated with cycloheximide and protein harvests were collected and analyzed for relative MYC expression and half-life (n = 3). (C) MCF10A cells were subjected to starvation media for 24 hours, and then treated with 4OHT for 24 hours to induce MYC expression. Cells were stained for ethynyl-2′-deoxyuridine (EdU) incorporation, revealing that cells expressing WT- and K326R-MYC could stimulate proliferation to a similar extent and were significantly different than EV expressing cells (n = 3, ***p<0.001, one-way ANOVA with Bonferroni's post-test). (D) MCF10A cells were starved for 24 hours and then treated with 4OHT and tunicamycin for an additional 24 hours. Cells were fixed and stained with propidium iodide and percent pre-G1 population was determined, revealing that both WT- and K326R-MYC could potentiate apoptosis to the same extent and significantly different compared to EV control (n = 3; *p<0.05, **p<0.01, one-way ANOVA with Bonferroni's post-test).

293Tv cells are routinely used successfully for mass spectrometry analysis and using this system we were able to identify a site of MYC SUMOylation. To address MYC biology, we have previously established a number of MYC-dependent model systems that allow us to score both loss and gain of function MYC mutants, including MCF10A cells [21]. Specifically, we have previously used this system to investigate the phenotype of a six-lysine mutant of MYC [22], as well as several MYC phosphomutants [23]. We sought to determine if K326R-MYC conferred a phenotype in this system. MCF10A cells were infected to generate 4-hydroxytamoxifen (4-OHT) inducible MYC cell lines. Cells were harvested for protein expression after 24 hours of 100nM 4-OHT treatment, revealing that both wild-type (WT) and K326R-MYC were expressed to a similar level (Fig. 3A). We first assessed if there was any difference in protein half-life between WT- and K326R-MYC. Using cycloheximide to inhibit protein synthesis, we immunoblotted for the levels of MYC following treatment revealing a non-significant trend (p = 0.07, n = 3) towards decreased half-life with the K326R-MYC mutant as compared to WT-MYC (Fig. 3B). We also confirmed that K326R-MYC retained interaction with MAX (S2A Figure).

We then tested if K326R-MYC was capable of driving cellular proliferation in the absence of growth factors in MCF10A cells. MCF10A cells were starved of growth factors for 24 hours to induce a G1 arrest of greater than 80% [22]. Cells were treated with 100 nM 4-OHT and 5-ethynyl-2′-deoxyuridine (EdU), a thymidine analog, for an additional 24 hours, and analyzed for EdU incorporation (Fig. 3C). Analysis revealed that WT- and K326R-MYC induced an average of 57±4% and 58±6% of cells to synthesize DNA, respectively, while vehicle treatment alone resulted in 8±6% positive cells, suggesting that SUMOylation of K326 does not play a role in MYC-induced cell cycle entry.

MYC can also potentiate cells to undergo cell death in response to antiproliferative signals [30], [31]. We thus also assessed the ability of WT- and K326R-MYC to drive cell death in MCF10A cells. Cells were serum-starved for 24 hours to induce a G1 arrest, then treated with 100 nM 4-OHT and 500 ng/mL tunicamycin for an additional 24 hours. Cells were harvested and stained with propidium iodide to assess the percentage of pre-G1 positive cells (Fig. 3D). Analysis revealed that both the WT- and K326R-MYC proteins potentiated cell death to the same extent, as compared to empty vector cells. Therefore, there is no significant difference between WT- and K326R-MYC to potentiate proliferation and apoptosis in MCF10A cells.

### The K326R Mutant Is Functionally Equivalent to WT-MYC in SH-EP Neuroblastoma Cells

**Figure 4:**
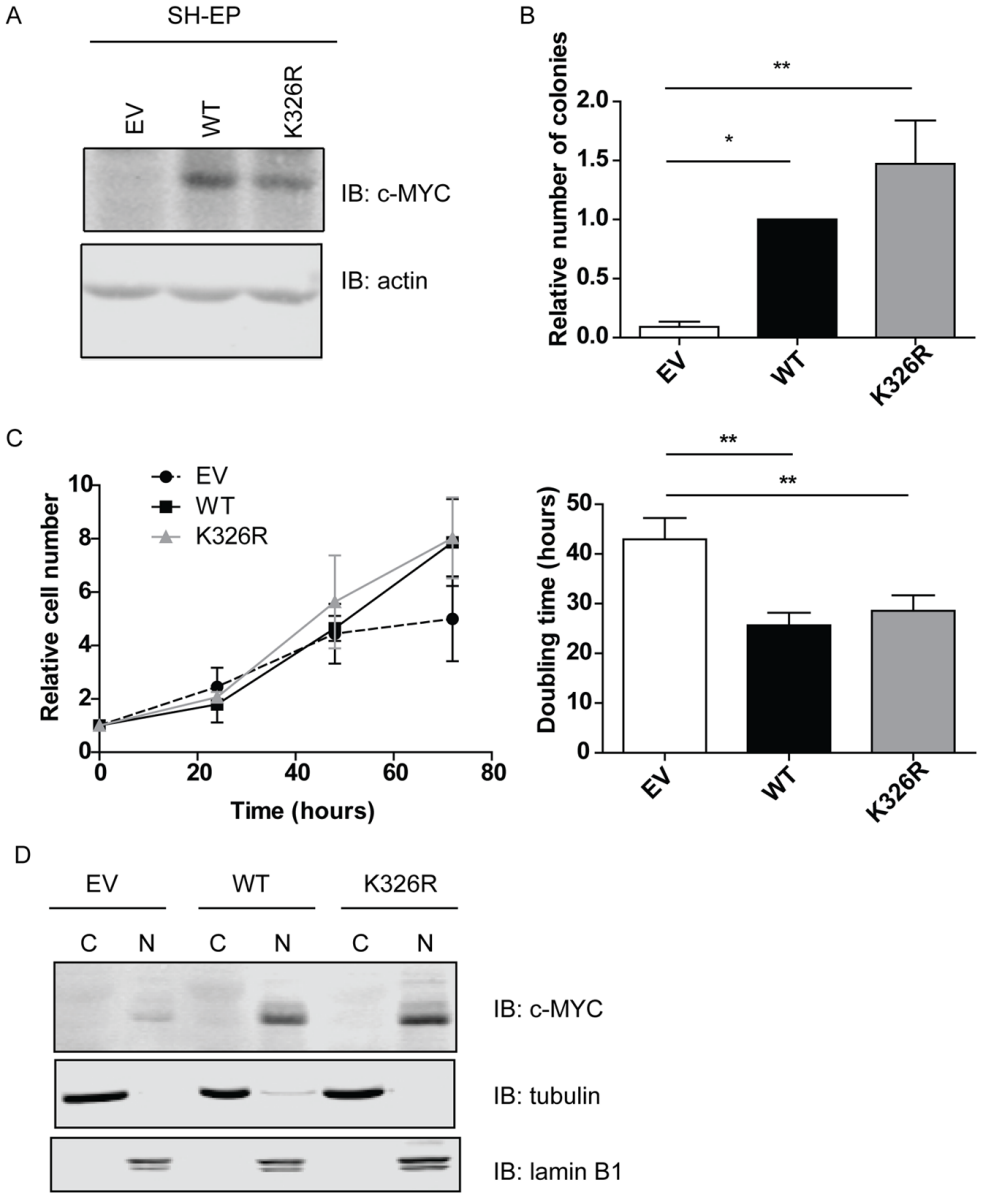
K326R mutant has no observed phenotypic effect in SH-EP cells. (A) Western blot depicting relative expression levels of EV control, WT-MYC and K326R-MYC. Actin was used as a loading control. (B) Soft agar colony formation assay revealed no significant difference between the ability of WT and K326R MYC to potentiate colony formation (n = 4; *p<0.05;**p<0.01; one-way ANOVA with Bonferroni post-test). (C) Left panel: Proliferation assay was performed demonstrating that EV is compromised in activity compared to WT- and K326R-MYC (n = 3). Right panel: Doubling time for EV, WT and K326R expressing cells. Both WT- and K326R-MYC had significantly different doubling times than EV control (n = 3, **p<0.01, one-way ANOVA with Bonferroni post-test). (D) Cellular fractionations revealed that WT- and K326R-MYC both localize to the nuclear compartment. Lamin B1 was used as a marker of the nuclear compartment (N), and tubulin was used as a marker of the cytosolic compartment (C).

We have previously established a number of MYC-dependent model systems that allow us to score both loss and gain of function MYC mutants [21]–[23]. One of these systems is SH-EPTET21/N-MYC (SH-EP) neuroblastoma cells, which have a doxycycline-regulated N-MYC allele that is repressed in the presence of doxycycline (S3 Figure). This system allows us to study the phenotype of MYC mutants in the presence of low levels of endogenous N-MYC and c-MYC proteins. WT-MYC, K326R-MYC or empty vector (EV) were introduced by retroviral infection and immunoblotted for expression following doxycycline treatment for 24 hours, demonstrating similar expression levels of both WT- and K326R-MYC (Fig. 4A). We also confirmed that both WT- and K326R-MYC retained interaction with MAX in SH-EP cells (S2B Figure). We then performed soft agar colony formation assays and found that WT-MYC and K326R-MYC could potentiate colony formation to a similar extent, as compared to EV control cells (Fig. 4B). To determine the effect of K326R on proliferation, SH-EP cells were seeded subconfluently, and DNA content was monitored over a 4-day period. Both WT- and K326R-MYC could potentiate proliferation over EV control cells (Fig. 4C, left panel) and significantly reduced the doubling time of SH-EP cells from an average of 43±4 hours to 26±3 and 29±3 hours respectively (Fig. 4C, right panel). As the SUMOylation site we identified is located in the nuclear localization signal (NLS) of MYC, we performed subcellular fractionation experiments to determine the localization of K326R-MYC. We found no difference in localization between WT- and K326R-MYC as both proteins were localized to the nuclear compartment (Fig. 4D). We conclude that WT- and K326R-MYC are functionally similar in SH-EP cells.

### WT- and K326R-MYC Activate and Repress Luciferase Target Gene Promoters to a Similar Extent

**Figure 5:**
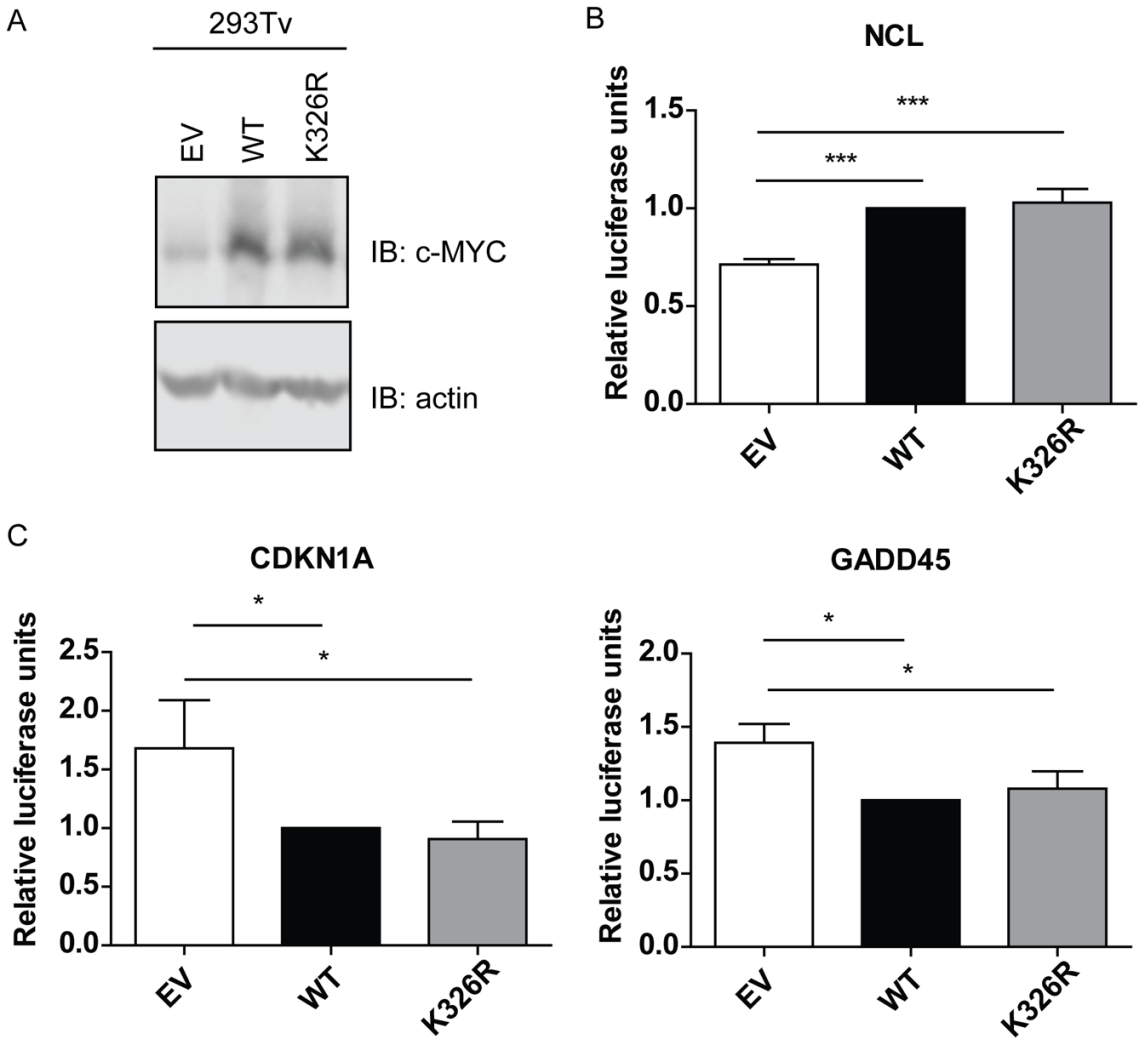
K326R mutant drives transcriptional activation and repression to the same extent as WT-MYC. (A) Western blotting demonstrated the expression of WT- and K326R-MYC following 24 hours of treatment with 100 nM 4OHT. Actin was used as a loading control. (B) Dual luciferase assay for nucleolin (*NCL*), an activated MYC target, revealed that both WT and K326R MYC can regulate its expression to the same extent (n = 3, p<0.001, one-way ANOVA with Bonferroni post-test). (C) Dual luciferase assays for growth arrest and DNA damage 45 (*GADD45*) and cyclin dependent kinase inhibitor 1A (*CDKN1A*), two MYC repressed targets, revealed that both WT- and K326R-MYC repressed these targets to the same extent (n = 3, *p<0.05, one-way ANOVA with Bonferroni post-test).

As a transcription factor, MYC can both activate and repress target genes [2], [32], [33]. To address whether K326R-MYC is altered in transcriptional activity we performed luciferase reporter assays. 293Tv cells were infected with lentiviral constructs to generate 4-OHT inducible MYC alleles. Cells were treated with 100nM 4-OHT for 24 hours and lysates were immunoblotted for MYC and actin, revealing similar expression levels between WT- and K326R-MYC expressing cells (Fig. 5A). 293Tv cells were seeded at 60% confluence, then transfected with constructs encoding MYC target promoters upstream of firefly luciferase as well as renilla luciferase controls and treated with 100 nM 4-OHT to induce MYC expression for 24 hours. Both WT- and K326R-MYC induced luciferase expression to the same extent when under the control of the nucleolin (*NCL*) promoter (Fig. 5B), and repressed luciferase expression to the same extent when downstream of the growth arrest and DNA damage 45 (*GADD45*) or cyclin-dependent kinase inhibitor 1A (*CDKN1A*) promoter (Fig. 5C). Our data thus suggest that K326R mutation does not alter the ability of MYC to activate or repress target genes.

### WT- and K326R-MYC Exhibit Similar Subcellular Localization

**Figure 6:**
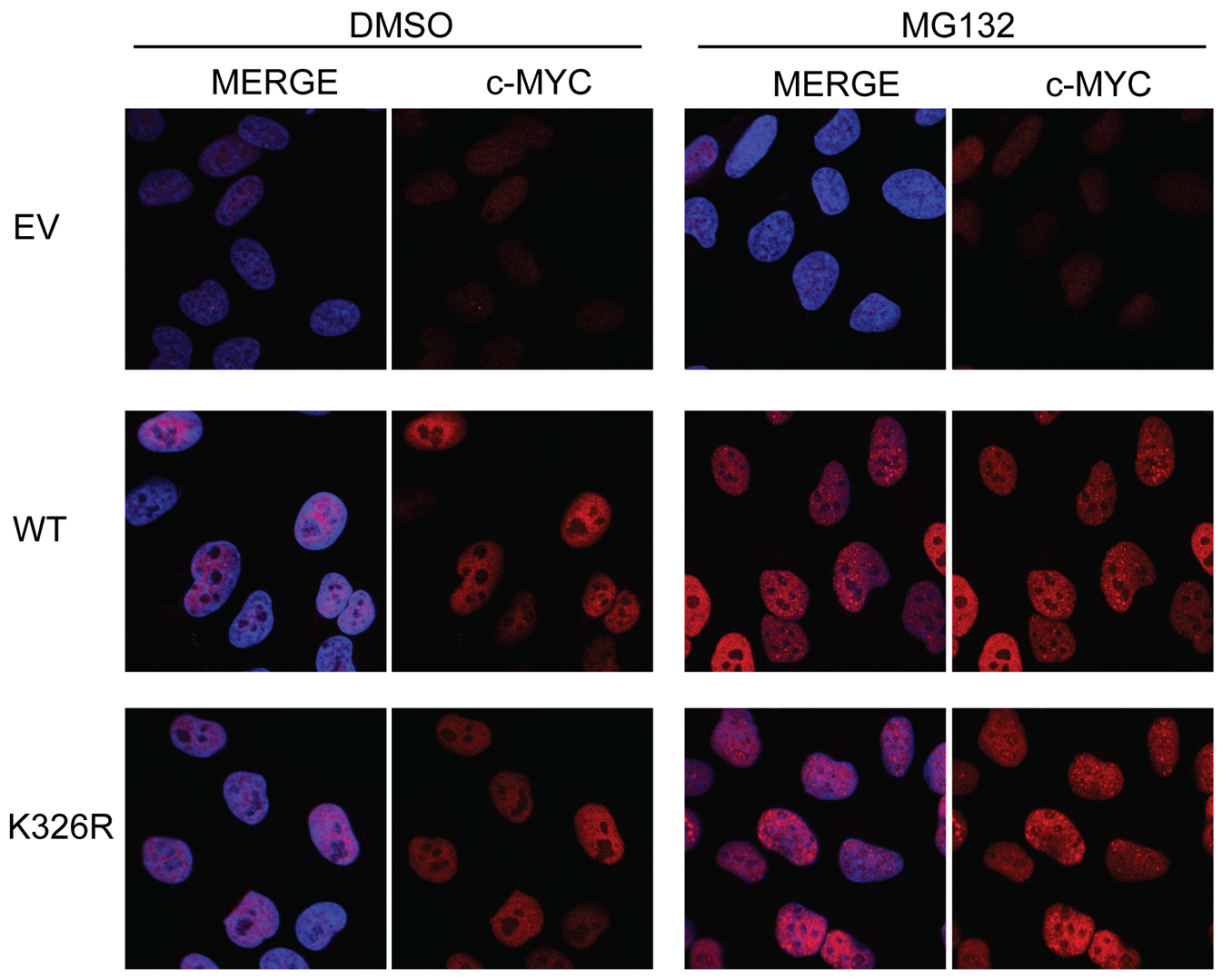
WT- and K326R-MYC exhibit similar subcellular localization. SH-EP cells were stained for MYC localization with either DMSO (vehicle) treatment or MG132 treatment for 4 hours. MYC signal is shown in red and nuclei in blue (DAPI). Empty vector expressing cells had minimal signal for MYC due to low endogenous expression of MYC in this cell line. Ectopic expression of WT- and K326R-MYC revealed nuclear signal. This signal was retained in the nucleus following proteasomal inhibition but gained a punctate staining pattern.

SUMOylation has previously been reported to affect subcellular localization of conjugated substrates [34]. To determine if SUMOylation at K326 could have an effect on the subcellular localization of MYC, immunofluorescence was conducted in SH-EP cells (Fig. 6). Subconfluent cells were treated with either DMSO or MG132 for 4 hours and then fixed and stained for MYC. Cells were counterstained with DAPI to visualize nuclei. Cells expressing EV control had minimal staining for MYC as this cell line expresses low levels of endogenous MYC. With transgene expression of WT or mutant MYC, there is increased signal for nuclear-localized MYC. Upon proteasome inhibition, the signal for MYC became punctate but remained nuclear, as previously described [35]. This observation was also confirmed in MCF10A cells (S4 Figure). We were therefore unable to detect any discernible difference in subcellular localization between WT and mutant MYC under normal or proteotoxic conditions.

### K326R-MYC Retains SUMOylation

**Figure 7:**
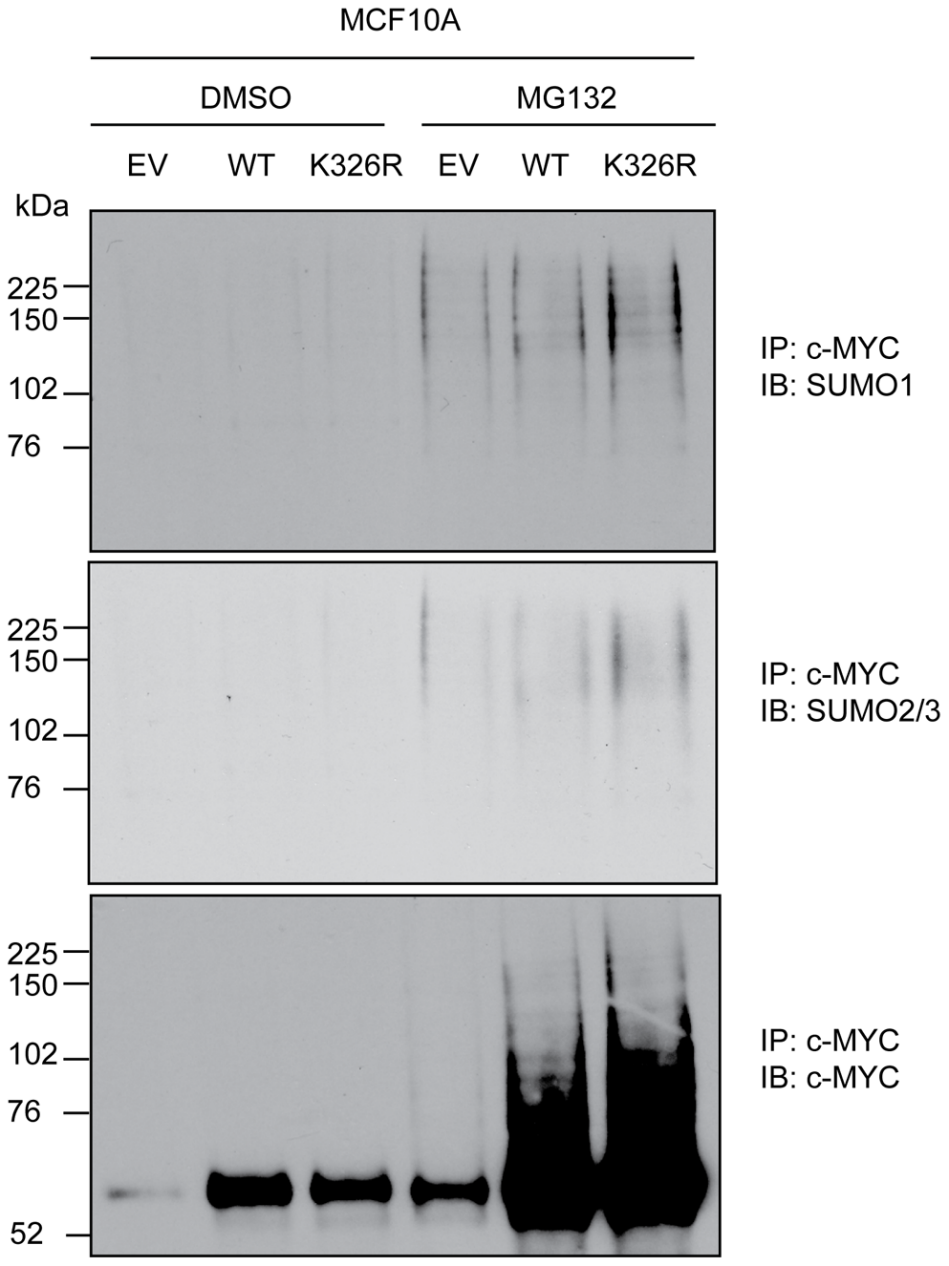
K326R-MYC retains SUMOylation MCF10A cells were treated with 4OHT for 24 hours to stimulate transgene expression. Cells were then either treated with vehicle (DMSO) or MG132 for 4 hours prior to harvest and immunoprecipitation for MYC. Immunoblotting was performed for SUMO1 and SUMO2/3 revealing that SUMOylation of MYC was enhanced following proteasomal inhibition. This effect was independent of the amount of MYC present in the pulldown (lower panel).

To determine if there may be additional sites of SUMOylation on MYC, we performed immunoprecipitation experiments in MCF10A cells. Cells were either treated with DMSO vehicle or MG132 for 4 hours. They were then immunoprecipitated for MYC, and immunoblotted for SUMO paralogs 1–3 (Fig. 7). As also shown in Fig. 1A, MG132 treatment resulted in visible signal for SUMOylated MYC in all cells treated with MG132. Furthermore, this was irrespective of the amount of MYC immunoprecipitated in the treated cells. This cannot be simply explained by MYC accumulation in response to MG132 treatment as similar levels of MYC were pulled down in DMSO treated cells expressing MYC transgenes and empty vector cells treated with MG132, suggesting this modification is readily detectable in response to proteotoxic stress. MYC SUMOylation was observed for endogenous MYC (EV) as well as ectopic WT and K326R MYC. This phenomenon was unchanged with the single K326R mutation suggesting the existence of other MYC SUMOylation sites.

## Discussion

Recent publications underscore the many diverse roles of the SUMO system in cancer. For example, SUMOylation of transcription factor AP-2 alpha (TFAP2A) has been reported to maintain the basal breast cancer phenotype, and inhibition of SUMOylation induces a basal to luminal transition through deregulation of TFAP2A transcriptional targets [36]. Furthermore, immunohistochemical data from invasive breast cancers suggested that a positive nuclear signal for the SUMO E2 (Ubc9, UBE2I), and the SUMO E3 protein PIAS1, is associated with good prognostic characteristics [37]. A recent paper also implicated a genetic interaction between MYC and the SUMO pathway in cellular transformation [16]. As SUMO and c-MYC both have intimate roles in transcriptional regulation and transformation, this study sought to uncover whether MYC could directly be SUMOylated. Using mass spectrometry we provide the first direct evidence of MYC SUMOylation with a high confidence SUMOylation site at K326. This site follows the consensus sequence for SUMOylation, ΨxKE/D, where Ψ is any hydrophobic amino acid.

Many transcriptional regulators have been reported to be SUMOylated, including MYB, androgen receptor (AR), p300, CREB, and p53 [15], [38]–[42]. SUMOylation of transcription factors frequently alters their transcriptional activity, as in the case of MYB, where transcriptional output is critically dependent on conjugation of SUMO1 at two conserved SUMOylation sites [15]. In our study, we did not identify an effect on transcriptional output by the K326R-MYC mutant, suggesting that signaling through this single residue is insufficient to alter MYC transcriptional activity.

It is possible that SUMOylation of MYC acts in conjunction with other PTMs in order to exert an overt phenotype. For example, we have previously reported that a larger 6KR six lysine-to-arginine mutation in this region (K298, K317, K323, K326, K341, K355) has greater transforming activity than WT-MYC in a number of different MYC-dependent assays both in tissue culture and mouse xenograft analysis [22]. Mechanistically, this was attributable to a gain in transcriptional activity that manifested as a result of loss of negative regulatory signaling through these residues [22]. Previous reports have identified K317 and K323 acetylation [43], [44] and K323 and K355 ubiquitylation [28] in this region. This study identifies K326-SUMOylation in this region as another potential negative regulator of MYC activity, possibly contributing to the phenotype of the 6KR mutant. Future studies will need to be performed to understand the interplay of signaling in this region.

Herein, we provide definitive mass spectrometry data showing MYC is directly SUMOylated, which builds on an independent study conducted concurrently with our research that was suggestive of MYC SUMOylation at K323 and/or K326 [18]. The latter study demonstrated that c-MYC and N-MYC shift in molecular weight in response to ectopic SUMO transfection, which could be partially rescued by mutation of K323 and K326. Our study further supports this finding and provides direct evidence, through mass spectrometry, that MYC can be SUMOylated at K326. In agreement with the report from Sabo *et al.*, we also find that SUMOylation of MYC is at relatively low abundance in asynchronously growing cells and can increase in abundance in response to proteasomal inhibition.

SUMOylated MYC is difficult to detect in growing cells and SUMOylation was still detected with the K326R mutant, thereby raising the possibility of additional yet unidentified SUMO sites on MYC. These additional sites may be context-dependent and require specific cellular stimuli to be robustly identified. This is not unusual, as SUMOylation of proteins is frequently associated with cellular stressors, such as a global increase in SUMOylation following heat shock [10] or the induction of SUMO proteins following viral infection [11]. Our mass spectrometry data also identified SUMO2/3 peptides upon SUMO1-Flag immunoprecipitation, raising the possibility of additional sites of SUMOylation, or the possibility of multi-SUMO chains. Identifying and characterizing the existence of additional sites of SUMOylation will be an important next step to understanding the functional role of MYC SUMOylation.

## Conclusions

In this study, we sought to determine whether MYC is directly SUMOylated. Using mass spectrometry, we have identified a SUMOylation site at K326, and have assessed a lysine to arginine mutant of this site for functional relevance. Abrogation of signaling through this residue has no effect on proliferation in two cell line models of MYC-dependent biology. Furthermore, no effect was seen on MYC-potentiated cell death in MCF10A cells, on soft agar colony formation in SH-EP cells or on dual luciferase reporter assays in transfected 293Tv cells. Overall we conclude that MYC can be SUMOylated at K326, however the K326R mutant is functionally similar to WT-MYC. Future studies will be needed to address the possibility of additional sites of MYC SUMOylation, and to identify the stimuli, proteases and conjugating proteins involved in the MYC SUMOylation cascade.

## Supporting Information

S1 Figure SUMmON screen shot of K326 SUMOylation on MYC. SUMmOn analysis indicates predicted and observed fragment ions for both the peptide modification (in this case SUMO-1, top) and the target peptide (MYC residues 325–331, bottom). SUMmOn-assigned dependent (dep or B′) and independent (Ind, or Y) fragment ions of the indicated charge series are highlighted in red and blue, respectively.
(TIF)

S2 Figure K326R-MYC retains interaction with MAX. (A) MCF10A cells were treated with 4OHT for 24 hours to induce transgene expression. Lysates were harvested and subjected to immunoprecipitation with an anti-MAX antibody or IgG control. Immunoblotting demonstrates the enrichment of signal for MYC in lanes corresponding to endogenous MYC (EV) and ectopic constructs (WT or K326R) as compared to IgG control. (B) SH-EP cells were treated with doxycycline for 24 hours to repress the expression of N-MYC. Cells were harvested and immunoprecipitated for MAX and immunoblotted for MYC. There is an enrichment of signal for both WT- and K326R-MYC as compared to IgG controls.
(TIF)

S3 Figure N-MYC expression in SH-EP neuroblastoma cells. SH-EP cells were treated with varying concentrations of doxycycline and immunoblotted for N-MYC expression. Tubulin was used as a loading control.
(TIF)

S4 Figure WT- and K326R-MYC display similar subcellular localization in MCF10A cells. MCF10A cells were treated with 4OHT for 24 hours to induce transgene expression. Cells were stained with anti-MYC antibody (red) and DAPI to mark nuclei (blue). Cells treated with MG132 displayed punctate MYC staining (both WT and K326R).
(TIF)
