# Supplementary figures and images for: Identification of c-MYC SUMOylation by Mass Spectrometry
Source: PLoS One. 2014 Dec 18;9(12):e115337. doi: 10.1371/journal.pone.0115337 (PMC4270761; doi:10.1371/journal.pone.0115337)

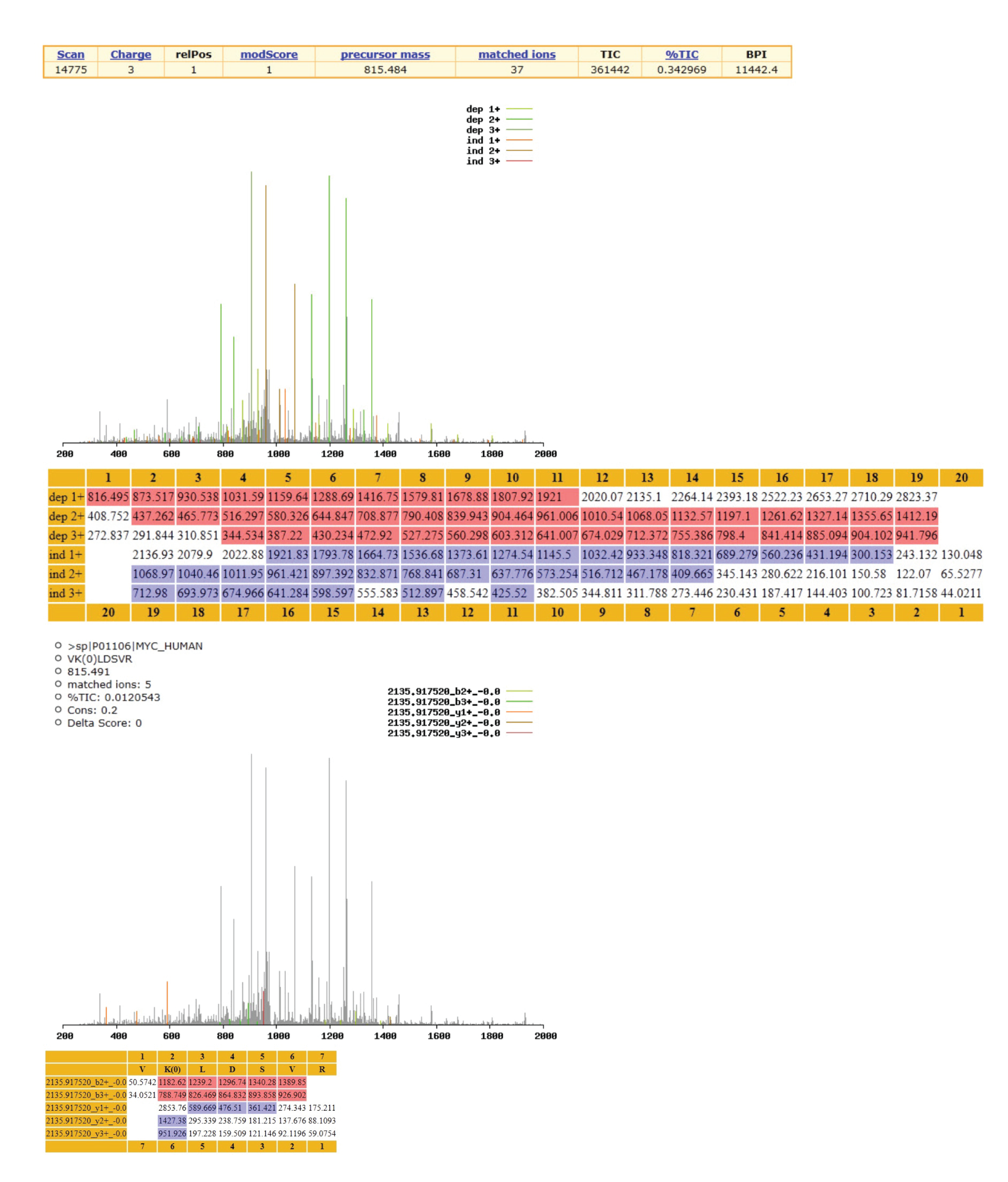

Supplement: S1 Figure — SUMmON screen shot of K326 SUMOylation on MYC. SUMmOn analysis indicates predicted and observed fragment ions for both the peptide modification (in this case SUMO-1, top) and the target peptide (MYC residues 325–331, bottom). SUMmOn-assigned dependent (dep or B′) and independent (Ind, or Y) fragment ions of the indicated charge series are highlighted in red and blue, respectively. (TIF) [file pone.0115337.s001.tif]

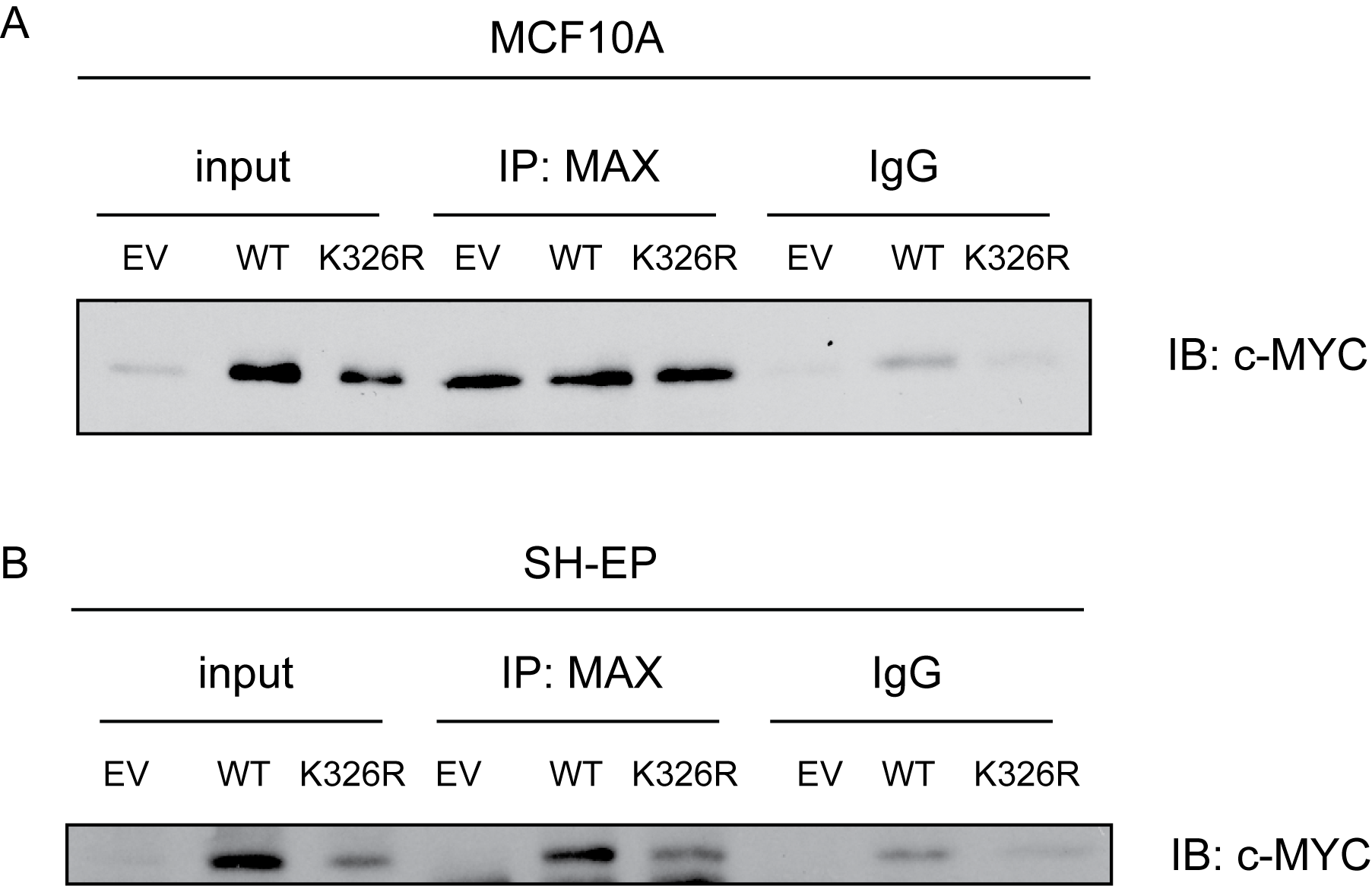

Supplement: S2 Figure — K326R-MYC retains interaction with MAX. (A) MCF10A cells were treated with 4OHT for 24 hours to induce transgene expression. Lysates were harvested and subjected to immunoprecipitation with an anti-MAX antibody or IgG control. Immunoblotting demonstrates the enrichment of signal for MYC in lanes corresponding to endogenous MYC (EV) and ectopic constructs (WT or K326R) as compared to IgG control. (B) SH-EP cells were treated with doxycycline for 24 hours to repress the expression of N-MYC. Cells were harvested and immunoprecipitated for MAX and immunoblotted for MYC. There is an enrichment of signal for both WT- and K326R-MYC as compared to IgG controls. (TIF) [file pone.0115337.s002.tif]

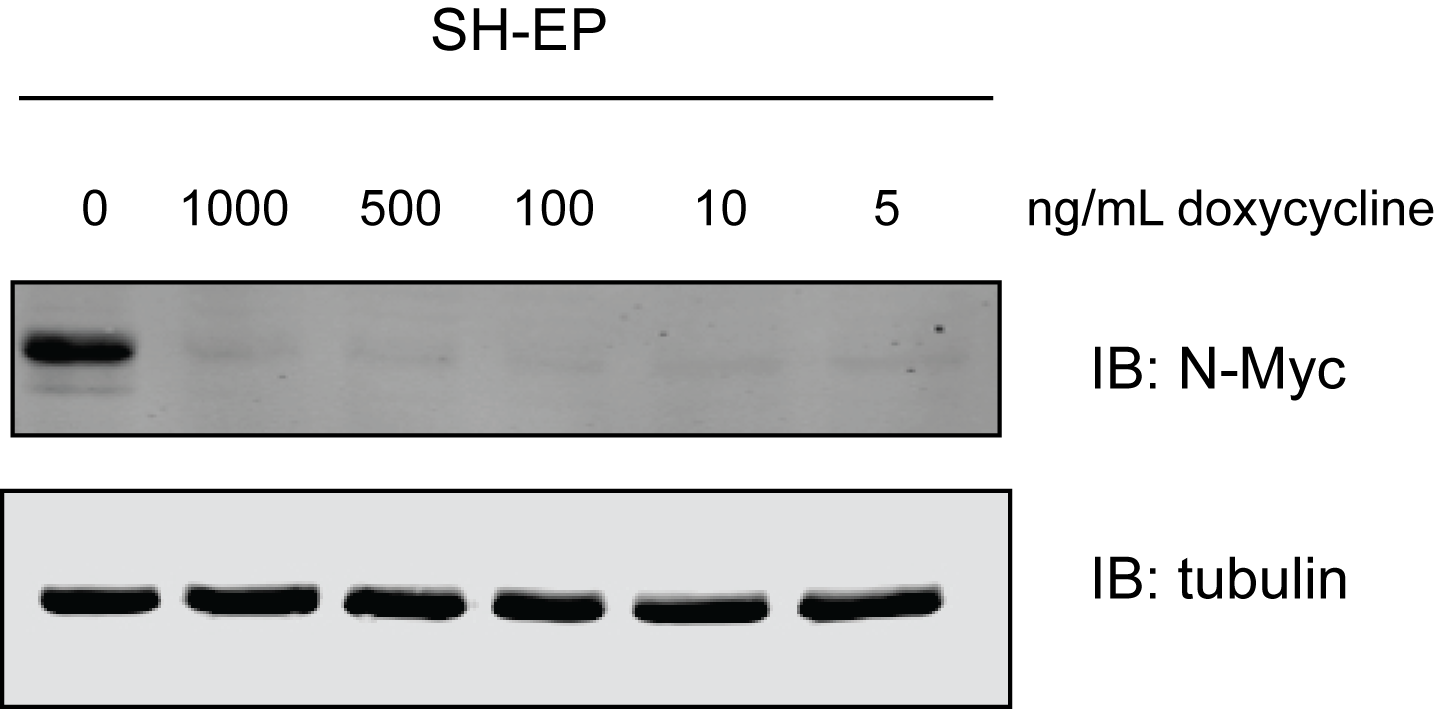

Supplement: S3 Figure — N-MYC expression in SH-EP neuroblastoma cells. SH-EP cells were treated with varying concentrations of doxycycline and immunoblotted for N-MYC expression. Tubulin was used as a loading control. (TIF) [file pone.0115337.s003.tif]

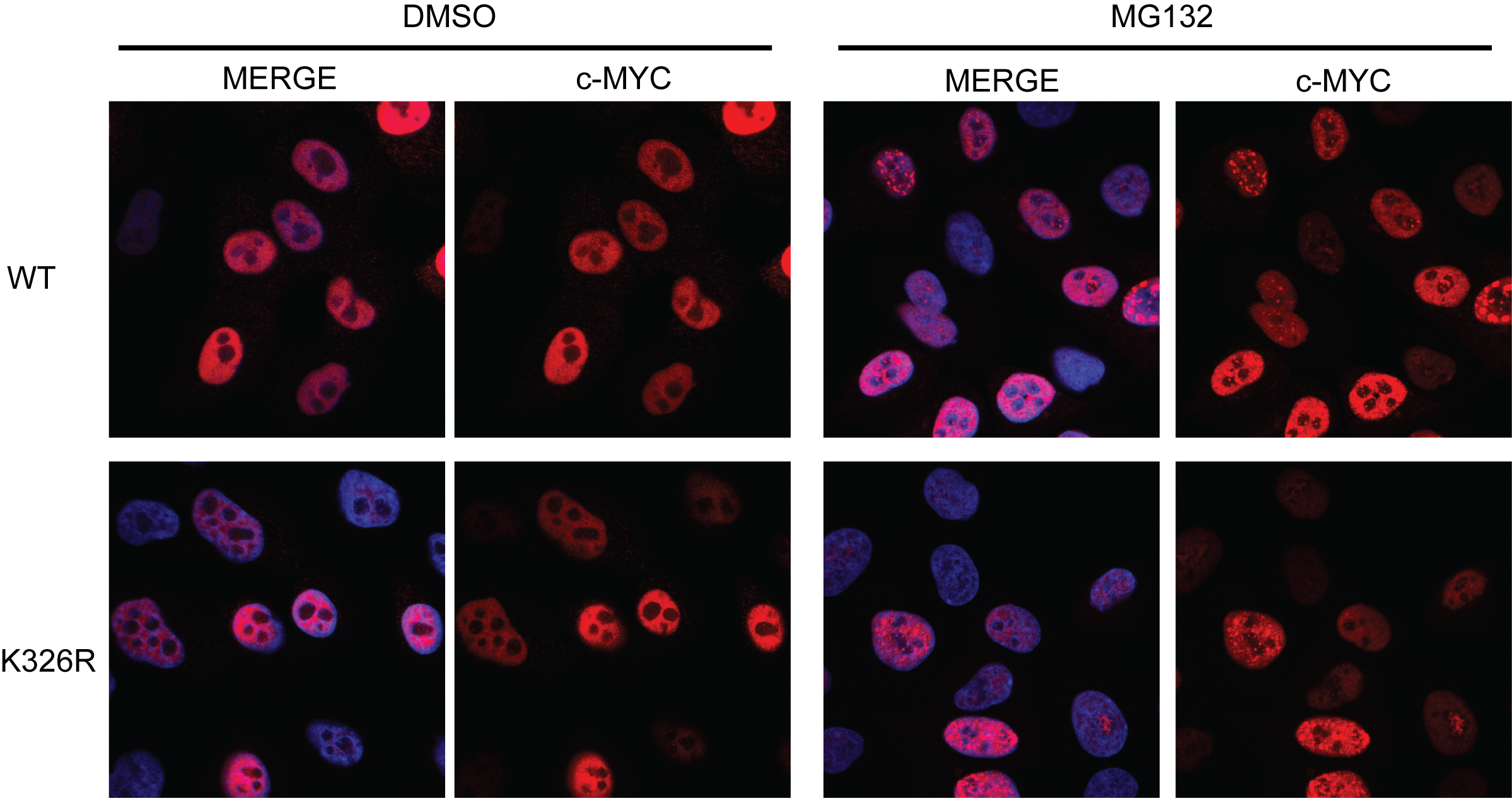

Supplement: S4 Figure — WT- and K326R-MYC display similar subcellular localization in MCF10A cells. MCF10A cells were treated with 4OHT for 24 hours to induce transgene expression. Cells were stained with anti-MYC antibody (red) and DAPI to mark nuclei (blue). Cells treated with MG132 displayed punctate MYC staining (both WT and K326R). (TIF) [file pone.0115337.s004.tif]
